# Citizens’ preference for e-participatory governance

**DOI:** 10.1371/journal.pone.0306268

**Published:** 2025-02-27

**Authors:** Oluwafemi Victor Oludu, Tuğberk Kaya, Damla Karagozlu

**Affiliations:** 1 Department of Management Information Systems, Cyprus International University, TRNC, Nicosia, Turkey; 2 Faculty of Business and Economics, Department of Business Administration, Rauf Denktas University, Nicosia, Turkey; Final International University: Uluslarasi Final Universitesi, TÜRKIYE

## Abstract

This paper investigates the preference, adoption, and utilization of e-government services among the citizens of Nigeria through preferred social media. Adopting a quantitative methodology approach with 1000 samples from various local governments and Statistically Analyzing the data, the research explores the extent to which Nigerian citizens have embraced e-government initiatives through social media and the factors influencing their adoption. Questions focusing on the availability of e-government platforms, social media e-governance, and e-participation level based on trust in government, the most preferred social media for e-participation were considered. Findings reveal varying levels of e-government adoption across different local government authorities, influenced by factors such as infrastructure limitations, institutional capacity, and political will, also a high level of participation through various social media platforms was observed, and the level of correlation between government trust and e-participation using the available platforms was found to be minimal. The paper contributes to the understanding of e-government implementation in the Nigerian context, offering insights into strategies for enhancing the effectiveness and sustainability of e-government initiatives at the local government level using social media Platforms.

## Introduction

In every sector, organization, and institution, proper communication is vital to relating with one another, and the applicability of proper communication not only fosters understanding and unity but also enhances the level of relationships, trust, and interactions among the parties involved in diverse ways [1,2]. The same principles apply to the government of any nation. Interactions and effective communication between citizens and government parastatals are inevitable. In any institution where proper communication is absent, there will be lots of issues, misunderstandings, and a lack of unity. There are several concepts of communication as a form of interchange of emotions, ideologies, thoughts, opinions, or even attitudes either through verbal or non-verbal mediums between two or more people [3]. Communication is not only limited to humans; most living things communicate. One of the major keys to communication is sending information and obtaining feedback, which is what makes it interactive and not a one-sided experience [4,5]. According to Berrett, (2006), if the government of a country and the leaders of a community of people neglect or violate the principle of proper communication, there will be chaos, miscommunication, and bad administration, and all of the aforementioned will be inevitable [5,6]. This is how the concept of communication relates to government, stakeholders, and citizens in a national setting. Proper communication based on good engagement is strongly encouraged to unify and promote synergy between the government (or ruler, as the case may be) and their populace [7]. Based on some facts stated by Ojenike et al 2014, [8] African countries especially Nigerians have lacked proper communication and interaction between the citizens and their respective governments which has limited the level of intended transparency aimed by the government. This in a way had voided the trust in the government of the citizens [9].

The recent advancement in technology has geometrically improved over the years, thereby causing numerous changes, ease, and enlightenment in different sectors, and one of the sectors of concern is the public sector. With the advancement and increment in the use of technology, there are vulnerabilities to these technologies that might endanger the adequate use of these technological advancements [10]. The term “e-government” has been applied in various contexts, encompassing a wide spectrum of meanings. It has been used to describe both the provision of online government services and the electronic exchange of information and services between the government, citizens, businesses, and other government entities [11]. Historically, e-government has primarily been understood as the utilization of information and communication technologies (ICTs) to enhance the effectiveness and efficiency of government agencies and facilitate the delivery of government services through digital platforms. The e-government system aims to enhance the government’s major operations positively to promote effective transparency and efficiency in governance [11–14].

government has become a revolutionary tool for managing public sector organizations worldwide. African governments have recognized the importance of information and communication technologies (ICT) and e-government in improving service quality and inclusive governance [15,16]. Many African sovereign governments are embracing e-government because it aids in the development of electronic platforms through which individuals gain access to public services and information via electronic means [17,18]. The United Nations e-government Survey serves as a benchmark to assess and compare countries’ e-government initiatives. The survey evaluates countries’ readiness for e-government and explores citizen engagement with e-government services [14,19]. According to the latest e-government statistics, Nigeria has one of the lowest e-government user numbers and adoption rates, with an index of 0.4525, ranking 140th out of 193 nations. Also, the e-participation level is low but a little bit better, with an index of 0.3068 and ranking 117th out of 193 nations [14,19]. The Local government forms the foundation basis of the governing bodies and extends to the state level and finally to the federal level. it, therefore, poses a concern if e-participation enhanced by e-government platforms has come to light at the local government level.

This brought the first aim of this research with an intent of investigating if citizens of Nigeria utilize e-government platforms from the local government level.

RQ1: Are there e-government platforms utilized by Nigerians from the local government level?

E-services have gained prominence in Nigeria to enhance public service delivery and citizen-government interactions. Studies have revealed some empirical evidence related to e-services in Nigeria and the increasing adoption of e-services among Nigerian citizens, with factors such as perceived usefulness, ease of use, and trust influencing adoption rates [20–22]. The implementation of e-services has shown positive impacts on tax administration, leading to improved efficiency, increased tax compliance, and revenue generation [23].

However, challenges are hindering the effective implementation of e-services in Nigeria. These include inadequate infrastructure, limited digital literacy, and bureaucratic bottlenecks. Overcoming these barriers is crucial for the success of e-service initiatives [21,22].

User perceptions and satisfaction with e-services are generally positive, but areas for improvement have been identified. Enhancing user interfaces, privacy and security measures, and responsiveness to user feedback are necessary steps for optimizing e-government services [22].

Overall, empirical evidence demonstrates the potential of e-services to transform public service delivery in Nigeria. Addressing challenges and incorporating user feedback will enhance the effectiveness and usability of e-services, ultimately leading to improved governance and citizen engagement.

Nigeria is in the western part of Africa with the highest population of over 200 million and has a total of 36 states and 774 local governments. Several attempts have been made to introduce and implement a working e-government in Nigeria but have failed due to barriers and limitations [24–26]. Irrespective of the barriers and limitations to the implementation of e-government in Nigeria, the level of e-participation has increased through evolving technology and social media use and spread throughout the country. According to Oyekan, (2022), Nigeria has a higher level of use of different social networking applications, which include Facebook, Twitter(X), TikTok, WhatsApp, Instagram, YouTube, etc. [27]. There is a migration in the ownership of Twitter at the time of this research and the name of the platform was changed to X, therefore for this paper, we will still refer to the platform as Twitter as it is popularly known by this name.

Considering the level of internet use and social media involvement in Nigeria as a whole, and with the geometric way in which technology and information systems are advancing, the level of citizen engagement and e-participation should be of concern, Since the outbreak of social media being a means of citizens engagement, it is pertinent to understand to which extent the social media has contributed to e-participation which could be a positive drive for the outburst of e-government spread in Nigeria. Advancements in the level of interaction and contributions from both sides of the government, stakeholders, and citizens through social media could easily attain an excellent level of effectiveness which brought the research questions 2 and 3.

RQ2: Has social media increased the level of e-participation and engagement?

RQ3: Is Twitter the most preferred means of e-participation?

This research aims to investigate the awareness, adoption, and use of the available e-participation platforms for e-government advancement at the local government level in Nigeria. Although some research has attempted to dive into the extent of e-government in Nigeria with the evolvement of social media governance [28–30], it is not enough to not understand the relationship between trust in governance and its correlation to e-participation which could be a contributing factor to the level of e-participation in Nigeria as a whole [31]. Given this, we considered the interrelationship between the level of trust in government and its impact on e-participation and verified the most preferred social media platforms that encourage more interaction and participation, especially from the government and its citizens.

RQ4: Does citizen’s trust in the government affect the level of e-participation?

Deducing the contribution of citizens’ trust to e-participation could be a determining factor in identifying some of the limitations of e-participation and e-government which has not been recently researched.

This research will propose a way forward to fill the communication gap and to properly highlight the most preferred and effective platform for e-participation, which will serve as a tool for the government to adopt and direct its energy toward improving its citizen engagement and, most importantly, increasing the level of e-government development soon through social media governance, as proposed by different authors [27–29].

## Literature review

The focus on e-government adoption and implementation in Nigeria has drawn the attention of researchers to a considerable extent. Different research has been conducted to advance the level of e-government adoption and possibly promote electronic participation among citizens at the local government level. An e-government initiative that has been implemented to improve the efficiency, transparency, and accountability of government processes is the National e-Government Strategy and Implementation Plan (NeGSP), which was launched in 2017 [30] and brought to light a level of e-government utilization. The NeGSP aims to provide a framework for the development and implementation of e-government initiatives across all levels of government in Nigeria. It identifies key areas for intervention, including the development of e-services, the establishment of a national data center, the implementation of a national identity management system, and the promotion of open data. Another important e-government initiative in Nigeria which was highlighted by [32,33] is the Treasury Single Account (TSA) system. The Federal Road Safety Corps (FRSC), which has developed an online platform for the issuance of driver’s licenses and vehicle registrations, which has reduced the time and cost required to obtain these documents is also a millstone achieved in e-government adoption as discussed by [34]. The National Identity Management Commission (NIMC) has also developed an online platform for the registration and issuance of national identity cards, which has improved the accuracy and reliability of identity data in Nigeria. With some of the proven evidence of the adoption of the e-government in Nigeria and with studies showing the low adoption rate of e-government in Nigeria [22,24], we propose the first Hypothesis,

*H1*: There is a significant presence and utilization of e-government platforms by Nigerians at the local government level.

Despite these achievements, there are still challenges to the effective implementation of e-government in Nigeria. One of the major challenges is the lack of adequate ICT infrastructure and connectivity, particularly in rural areas. This has limited the reach and impact of e-government initiatives in Nigeria and has hindered the participation of citizens in digital governance processes [35]. In addition, there is a need for greater collaboration and coordination among government agencies in the implementation of e-government initiatives, as well as a need for capacity building and skills development among government officials and citizens [36,37].

Other studies conducted on e-government in Nigeria have focused on various aspects of e-government implementation and its impact on government processes, citizen participation, and service delivery. Endong, and a few other authors [37–39] examined the challenges and prospects of e-government implementation in Nigeria. The study identified a lack of political will, inadequate ICT infrastructure and connectivity, poor data management, and inadequate funding as some of the major challenges facing e-government implementation in Nigeria. The studies recommended the need for sustained political commitment, capacity building, and skills development, and the establishment of a dedicated e-government agency to coordinate and oversee e-government initiatives in Nigeria.

The impact of e-government on citizen participation in Nigeria was examined and in this series of studies, it was pointed out that while e-government initiatives have the potential to increase citizen participation in governance processes, there are several barriers to their effective implementation, including limited access to ICT infrastructure, low levels of digital literacy, and a lack of trust in government institutions [35,40,41]. We assert the following hypotheses:

H2: There is a significant relationship between individuals’ level of trust in the government and their level of e-participation, with higher levels of trust leading to increased e-participation. Investment in ICT infrastructure and the development of user-friendly e-services, with the promotion of citizen engagement and participation in e-government processes, was discussed by [42] to be a way forward in enhancing citizen participation. It then surfaces that with the recent outburst of social media penetration among citizens, e-participation through social media has a high tendency to build the e-government initiative and adoption as seen by [43,44]

Ogunleye et al. [33] examined the factors influencing the adoption of e-government services in Nigeria. The study found that perceived usefulness, perceived ease of use, perceived risk, and trust were significant predictors of e-government adoption in Nigeria. The study recommended the need for the development of user-friendly e-services, the provision of adequate ICT infrastructure and connectivity, and the establishment of trust and credibility in e-government processes.

Alarabiat et al. [45] predicted a theoretical model that explores the factors influencing citizens’ acceptance of e-participation initiatives facilitated by social media. The authors propose a theoretical model that predicts citizen acceptance based on factors such as perceived usefulness, perceived ease of use, social influence, trust, and perceived risk. The study emphasizes the significance of social media in promoting citizen engagement and participation in government processes. By understanding these factors, governments can enhance the design and implementation of e-participation initiatives to encourage greater citizen acceptance and engagement.

Among other studies emphasizing the use of social media for interaction and engagement, Ozkent [46] places more emphasis on the dominance Twitter has over other social media platforms. He outlined the usefulness of publishing blogs as the most popular microblogging site. Due to the literature evidence of the popularity of Twitter(X) as the most common microblogging site, which is also used for publishing information, we therefore propose that,

H3: Twitter is the most preferred means of e-participation among citizens of Nigeria.

An Investigation of Social Media as a Government Digital Public Relations Tool [47] revealed the use of social media as a digital public relations tool by the Nigerian government. The study explores the benefits and challenges of using social media platforms for government communication and engagement with citizens. it highlights the importance of strategic planning, content management, and responsiveness in maximizing the effectiveness of social media as a government PR tool. The findings suggest that social media can enhance government-citizen interactions, improve transparency, and facilitate information dissemination. The study provides insights into the Nigerian context and offers recommendations for optimizing social media usage for government communication purposes. Hence, we propose that,

H4: The use of social media has significantly increased the level of e-participation and engagement among citizens.

With an extensive level of research already conducted on social media means of engagement and e-government, the place of citizens and individuals, and preference in the use of social media has not been extensively researched based on the literature. A few studies by [48–50] commonly investigate communication preferences and social media engagement through a survey-based approach, where each of the authors has a limited view of the available social media and the general audience. Most of the available recent research on social media was not detailed in focusing on the most common social media platforms and the general populace to which the discovery can be generally applicable to most fields and even a large population.

To establish the validity of the hypotheses, we developed a counter null and alternative hypotheses thereby giving a valid argument that,

Null Hypothesis: There is no correlation between the use of Twitter in a local government and the level of e-participation.

Alternative Hypothesis: There is an association between the use of Twitter in a local government and the level of e-participation.

There is an association between the use of social media in a local government and the level of trust from citizens toward their government.

There is no correlation between the use of social media in a local government and the level of trust from citizens toward their government.

With the level of research already conducted in Nigeria and in general, there are still several gaps to be filled regarding the development and optimum adoption of e-government opportunities, especially in the area of citizens’ preference, involvement, and participation in e-services and government initiatives. This research mainly focuses on the level of awareness, adoption, and participation in e-government in Nigeria and provides a valid argument for the preferred medium of e-participation from the local level in the development of e-government in the nation.

## Methods

### Study design

This study aims to understand the level of adoption and utilization of e-government in the Local government of Nigeria by considering the e-participation level of citizens through a preferred social media platform which could be affected by factors such as trust in government. In other to achieve this, we adopt a quantitative methodological research approach, with the processes of data collection, analysis, evaluation, and justification. Primary data was collected using a Google form questionnaire with a total of 1000 administered questionnaires containing 4 sections and a total of 35 closed-ended questions with a 5-point Likert scale structure. This question pattern was adopted to encourage ease of analysis, standardization, and time efficiency. Details on demographics and the variables in the research questions were considered in the design of the questionnaire to enable us to analyze the collected data considering the variables under investigation. The SPSS statistical application was used in the analysis of the data where Cronbach’s Alpha was used to test the reliability and factor analysis used to identify underlying relationships among the observed variables. In other to mitigate the common biases in questionnaires which can undermine the reliability and validity of the collected data, we adopted a pre-testing strategy that involves trialing the questionnaire with a small sample to identify and rectify ambiguities or biases. Also, we utilize clear and concise language to ensure respondent comprehension, randomizing question order and response options mitigates order effects bias. we tried to avoid leading questions and employing standardized Likert scales to minimize bias. we also ensure anonymity and confidentiality to foster honest responses, especially for sensitive questions prone to social desirability bias. Finally, pilot testing with a small group was first carried out to allow for bias identification and questionnaire refinement before full-scale implementation.

### Sampling technique

The research was conducted by selecting all the 774 local governments in the 36 states of Nigeria and in a way to mitigate any form of bias and discrepancy of data, we employed the stratified random sampling technique where each of the states in the geopolitical zones was selected as strata [51]. 1000 Samples were randomly selected with an average of 27 selected representatives from different local governments in the state. In the situation where we could not get any representative respondent in the local government, we select more respondents from the same state to measure up for the least number required for each state. There was less focus on 2 geopolitical zones (north-east and north central) with a low turnout of respondents due to the high level of insecurity at the time of this data collection. The questionnaires were administered and filled in by participants from the various local governments in the geopolitical zones and collated over 4 months. This duration was due to the huge number of instrumentations that needed to be collated which needs proper monitoring and guidance in situations where respondents are unclear about the questions.

#### Inclusion and exclusion criteria

The age group of 1–18 years was excluded from the administration of the questionnaire due to the emotional imbalance and limitations of the age range from the use of mobile devices and public participation. Also, two geopolitical zones, north-central and northeast, had a low turn-out due to the insecurity in these regions at the time of the data collection. However, the situation may not have affected the online distribution of questionnaires but could have created biases in the minds of the respondents when responding to the questions. Furthermore, the four geopolitical zones south-west, north-west, south, and south-east had a better turnout and were considered to be more effective and reliable based on their exposure to information and communications technology (ICT) and social media (SM) engagement, which were reported to be higher in these regions [52].

## Results

The following findings were obtained after the analysis of the collected data. The collected data were analyzed using the SPSS statistical analysis tool. The data collected and analyzed show that 49.1% of the respondents were female, while 50.9% were male, and the age range. data show that 57.2% of the respondents’ ages were between 18 and 34 years, 32.6% were 35–50 years, and the other 10.2% were over 50 years. The mean age was in the range of 18–34 years. According to the respondents’ zoning data, 44.1% were from the south-west, 23% from the south-east, 14.6% from the north-west, 10.9% from the souths-south, 5.8% from north-central, and 1.6% from the north-east.

The data show that 8.1% of the respondents were neither interested in nor used any platform for e-participation, including social media, while 70.9.4% (N = 778) utilized social media for e-participation, and the remaining 21% were unsure of their participation using any platform. A total of 34.3% (N = 362) of the respondents declared a lack of awareness of any form of e-government platform, while 37.9% (N = 399) acknowledged the existence of e-government platforms in their LG and the others were indecisive. The analysis also reveals that 54.4% (N = 573) of the respondents acknowledged an interest in e-participation due to their trust in the government, while 14.6% (N = 218) lacked willingness due to their lack of trust in the government. The other variables considered and the results of the analysis of the respondents’ data are shown in Table 1 below.

**Table 1:** Data analysis of respondents.

	Descriptive Analysis of Variables					
Variables	Acceptance		Neutral		Rejection	
	n	%	n	%	n	%
**Interest in SM e-Part**	778	77.8	40	4.0	182	18.2
**Prefer SM for e-Part**	763	76.3	128	12.8	109	10.9
**Gov’t Trust as condition of e-Part**	573	57.3	218	21.8	209	20.9
**e-Gov Availability in LG**	293	29.3	308	30.8	399	39.9
**SM Penetration in LG**	575	57.5	207	20.7	218	21.8
**Prefer Twitter for e-Part**	590	59.0	161	16.1	249	24.9
**Prefer WhatsApp for e-Part**	737	73.7	123	12.3	153	15.3
**Prefer Facebook for e-Part**	611	61.1	190	19.0	199	19.9
**Prefer Telegram for e-Part**	480	48.0	187	18.7	332	33.2
**Prefer Instagram for e-Part**	479	47.9	192	19.2	329	32.9
**Prefer other SM for e-Part**	219	21.9	111	11.1	670	67.0

NB: N = 1000, i.e., the number of respondents.

The reliability of the questionnaire was tested using Cronbach’s Alpha whereby the alpha value of the questionnaire should be between the values 0.9 and 0.6 for acceptability. Here, the alpha value is 0.723, (Table 2) which implies that the questionnaire is reliable.

**Table 2:** Cronbach’s Alpha to measure the reliability of the questionnaire.

**Reliability Statistics**		
**Cronbach’s Alpha (α)**	Cronbach’s Alpha Based on Standardized Items	No. of Items
0.734	0.723	28

NB: 0.8 > α ≥ 0.7 is acceptable.

Cronbach’s alpha for measuring reliability can be as high as 0.9, which is regarded as the best acceptable value, and can also be as low as 0.5 or less, which is considered to be very poor and unacceptable.

The use of social media has been argued to affect the level of e-participation among citizens in the selected LG.

**Fig 1:**
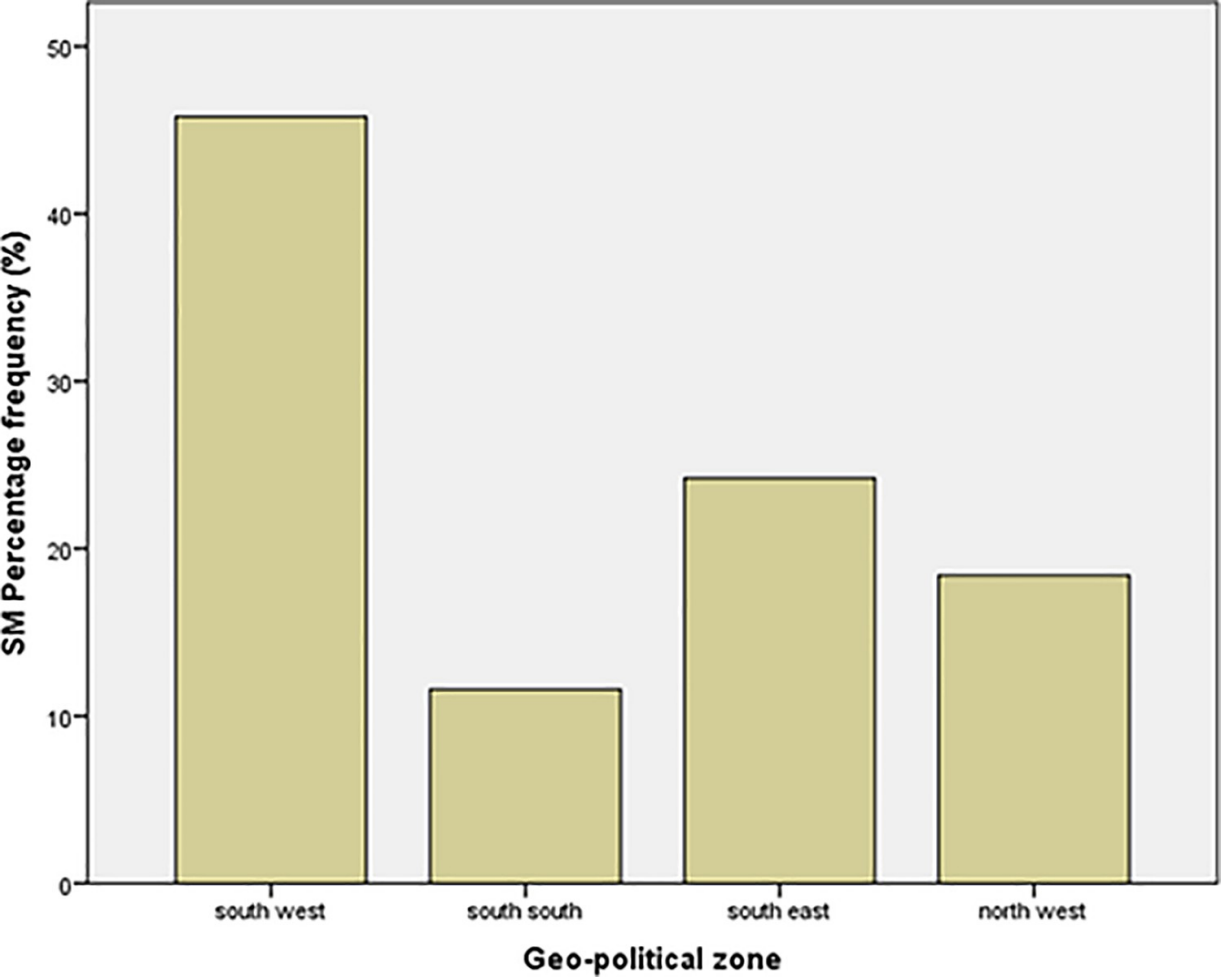
Percentage frequency of social media (SM) users for e-participation in the selected geopolitical zones.

**Fig 2:**
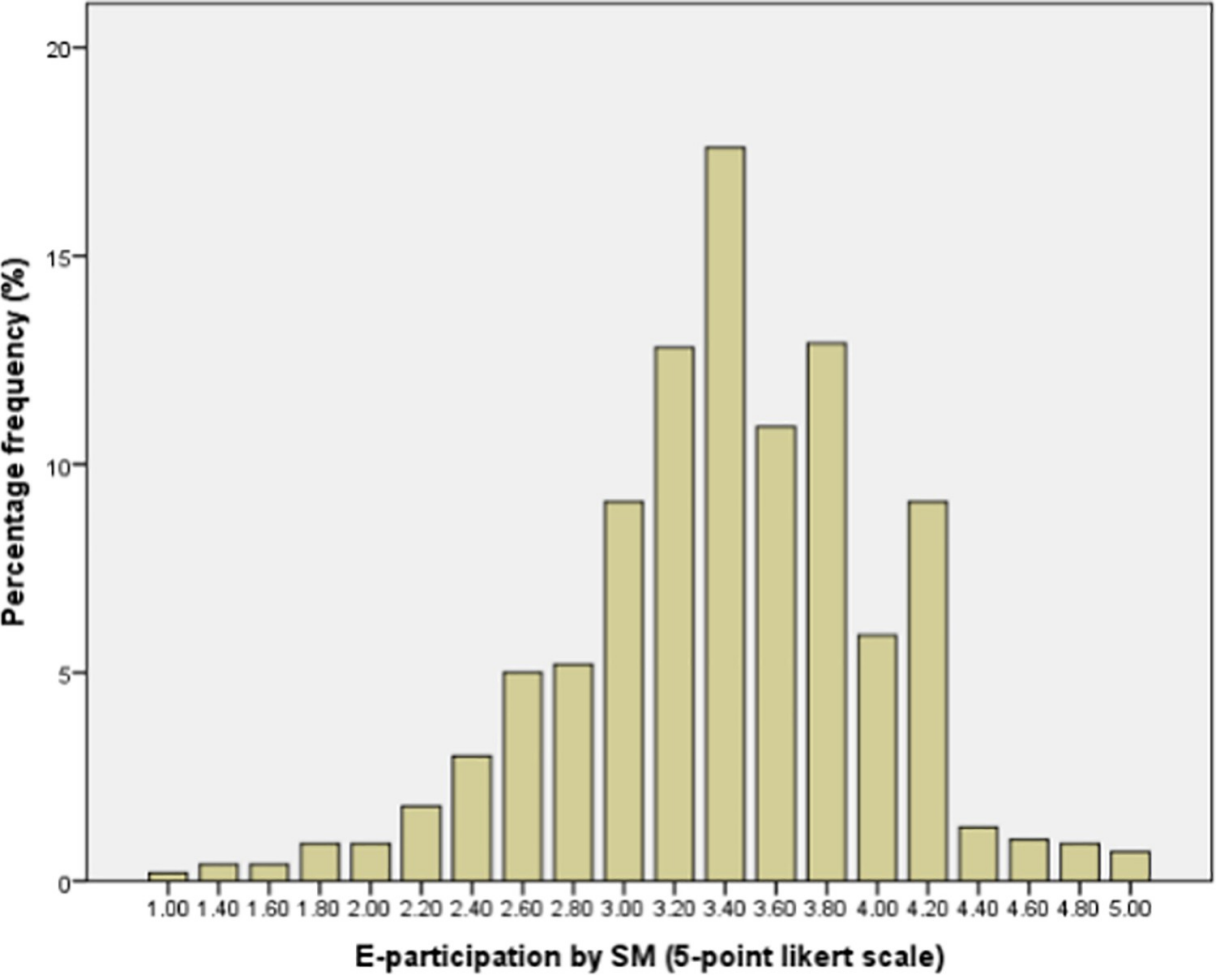
The skewness of the percentage frequency of the mean is based on the Likert scale of social media users for e-participation.

In this study, Fig 1 shows the number of social media (SM) users channeled toward e-participation in the selected geopolitical zones and Fig 2 shows the skewness of the percentage frequency of the mean which is based on the Likert scale.

The actual value of skewness shown below (Table 3) is −0.417, which implies that the distribution is fairly symmetrical. A skewness value that lies between 1 and 0.5 or −0.5 and −1 can be said to be moderately skewed, while a value between −0.5 and 0.5 is said to be fairly symmetrical.

**Table 3:** Skewness and deviation of the e-participation on social media.

	Gender	Age	E-Participation on SM	Geopolitical Zone	
**N**	Valid	1000	1000	1000	1000
	Missing	0	0	0	0
**Mean**	1.50	1.55	3.3986	2.15	
**Std. Deviation**	0.500	0.678	0.61261	1.189	
**Skewness**	−0.008	0.837	−0.417	0.363	
**Std. Error of Skewness**	0.077	0.077	0.077	0.077	

The skewness value lies between 0 and −0.5 as seen in Fig 2, depicting that the distribution is moderately skewed and fairly symmetrical (skewness is the measurement of how symmetrical a distribution is, which explains how symmetric, or identical the right and left sides of the graph are with each other).

The standard deviations between the means of the variables e-participation and government trust are shown in Table 4 below.

**Table 4:** Standard deviations of e-participation and government trust.

Descriptive Statistics					
	N	Minimum	Maximum	Mean	Std. Deviation
**E-participation via SM**	1000	1.00	5.00	3.3986	0.61261
**Govt. trust**	1000	1	5	3.56	1.221
**Geopolitical zone**	1000	1	4	2.15	1.189
**Valid N (listwise)**	1000				

The value of the standard deviation for e-participation is 0.6 which implies that the sample mean of 3.4 is within 0.6 of the actual population mean. This also virtually means that about 20% of the normally distributed data lie between the mean and 0.6 standard deviations to the right of the mean.

Tables 4 and 5 show the standard deviation of gender, age, employment, geopolitical zone, Twitter users, e-participation, and government trust.

**Table 5:** Standard deviation of gender, age, employment, geopolitical zone, Twitter users, e-participation, and government trust.

Descriptive Statistics					
	N	Minimum	Maximum	Mean	Std. Deviation
**Gender**	1000	1	2	1.50	0.500
**Age**	1000	1	3	1.55	0.678
**Employment**	1000	1	3	1.91	0.769
**Geopolitical zone**	1000	1	4	2.15	1.189
**Govt. trust**	1000	1	5	3.56	1.221
**E-participation via SM**	1000	1.00	5.00	3.40	0.61261
**Twitter use by LG**	1000	1	5	3.36	1.317
**Valid N (listwise)**	1000				

### Factor analysis

Factor Analysis is the process of reducing the dimension of items in a sample where the number of variables is numerous. Here, a set of variables (24) is reduced to dimensions or super variables by loading a set of characteristic factors together. If the items load together on the same construct using a factor-loading element, this implies true correlation. In other words, this factor analysis will help determine the correlation between the numerous variables.

Using a statistical software package, the following were analyzed as shown below. Tables 6–10 show the correlation matrix between variables. Within the same variable, unity is singular (1.000), while the other values show the correlations between different variables.

**Table 6:** Correlation matrix between all variables.

	SM Use	Info Use	IT SM Use		SM as Evolving Tool	SM Use for LG ePart	Govt. Trust
**Correlation**	**SM use**	1.000	0.063	−0.219	−0.327	−0.262	−0.160
	**Info use**	0.063	1.000	0.073	0.030	0.018	0.109
	**IT SM use**	−0.219	0.073	1.000	0.594	0.467	0.170
	**SM as evolving tool**	−0.327	0.030	0.594	1.000	0.530	0.208
	**SM use for LG ePart**	−0.262	0.018	0.467	0.530	1.000	0.321
	**Govt. trust**	−0.160	0.109	0.170	0.208	0.321	1.000
		−0.458	0.011	0.412	0.452	0.411	0.240

**Table 7:** Correlation matrix between all variables.

		Inc eGov dev	Bad attitudes of leaders	SM penetration rate	SM types	Prefer Twitter	Prefer Facebook
**Correlation**		−0.085	−0.055	−0.105	−0.431	−0.131	−0.158
	**Inc eGov dev**	1.000	0.208	0.357	0.139	0.160	0.179
	**Bad attitudes of leaders**	0.208	1.000	0.072	0.204	0.001	−0.019
	**SM penetration rate**	0.357	0.072	1.000	0.187	0.227	0.237
	**SM types**	0.139	0.204	0.187	1.000	0.171	0.229
	**prefer Twitter**	0.160	0.001	0.227	0.171	1.000	0.222
	**Prefer Facebook**	0.179	−0.019	0.237	0.229	0.222	1.000

**Table 8:** Correlation matrix between all variables.

		Prefer Instagram	Prefer Telegram	Prefer WhatsApp	Another effective SM	Twitter use by LG	Facebook use by LG
		−0.049	−0.108	−0.252	0.328	−0.056	−0.177
**Correlation**		0.199	0.149	0.113	0.006	0.209	0.171
	**Prefer Instagram**	1.000	0.581	0.244	−0.069	0.320	0.242
	**Prefer Telegram**	0.581	1.000	0.311	−0.086	0.325	0.293
	**Prefer WhatsApp**	0.244	0.311	1.000	−0.065	0.186	0.263
	**Another effective SM**	−0.069	−0.086	−0.065	1.000	−0.015	−0.120
	**Twitter use by LG**	0.320	0.325	0.186	−0.015	1.000	0.251
	**Facebook use by LG**	0.242	0.293	0.263	−0.120	0.251	1.000

**Table 9:** Correlation matrix between all variables.

		Instagram use by LG	Telegram use by LG	WhatsApp use by LG	Use of Another SM by LG	SM Inc e-participation	More Effort made by LG
**Correlation**		−0.012	−0.062	−0.247	0.384	−0.281	−0.458
	**Instagram use by LG**	1.000	0.649	0.313	−0.022	0.031	0.004
	**Telegram use by LG**	0.649	1.000	0.343	0.008	0.081	−0.001
	**WhatsApp use by LG**	0.313	0.343	1.000	−0.136	0.272	0.297
	**Use of another SM by LG**	−0.022	0.008	−0.136	1.000	−0.154	−0.349
	**SM inc e-participation**	0.031	0.081	0.272	−0.154	1.000	0.523
	**More effort made by LG**	0.004	−0.001	0.297	−0.349	0.523	1.000

**Table 10:** Correlation between the variable groups.

Correlations								
		Gender	Age	Employment	Geopolitical zone	E-participation via SM	Twitter use by LG	Govt. trust
**Gender**	**Pearson Correlation**	1	0.019	0.062	0.033	0.011	−0.010	−0.029
	**Sig. (2-tailed)**		0.551	0.051	0.295	0.718	0.747	0.362
	**N**	1000	1000	1000	1000	1000	1000	1000
**Age**	**Pearson Correlation**	0.019	1	0.008	−0.186 **	−0.046	0.057	0.024
	**Sig. (2-tailed)**	0.551		0.797	0.000	0.143	0.072	0.445
	**N**	1000	1000	1000	1000	1000	1000	1000
**Employment**	**Pearson Correlation**	0.062	0.008	1	0.141 **	−0.010	−0.030	−0.101 **
	**Sig. (2-tailed)**	0.051	0.797		0.000	0.741	0.338	0.001
	**N**	1000	1000	1000	1000	1000	1000	1000
**Geopolitical zone**	**Pearson Correlation**	0.033	−0.186 **	0.141 **	1	0.101 **	−0.093 **	−0.116 **
	**Sig. (2-tailed)**	0.295	0.000	0.000		0.001	0.003	0.000
	**N**	1000	1000	1000	1000	1000	1000	1000
**E-participation via SM**	**Pearson Correlation**	0.011	−0.046	−0.010	0.101 **	1	0.066 *	0.231 **
	**Sig. (2-tailed)**	0.718	0.143	0.741	0.001		0.037	0.000
	**N**	1000	1000	1000	1000	1000	1000	1000
**Twitter use by LG**	**Pearson Correlation**	−0.010	0.057	−0.030	−0.093 **	.066 *	1	0.187 **
	**Sig. (2-tailed)**	0.747	0.072	0.338	0.003	.037		0.000
	**N**	1000	1000	1000	1000	1000	1000	1000
**Govt. trust**	**Pearson Correlation**	−0.029	0.024	−0.101 **	−0.116 **	0.231 **	0.187 **	1
	**Sig. (2-tailed)**	0.362	0.445	0.001	0.000	0.000	0.000	
	**N**	1000	1000	1000	1000	1000	1000	1000

* Correlation is significant at the 0.05 level (2-tailed).

** Correlation is significant at the 0.01 level (2-tailed).

Correlations above 0.8 are described as strongly correlated as this can lead to unstable estimates of the regression coefficients and inflated standard errors. Thus, the accepted range for correlation is between 0.00001 and 0.8. Any variable with a value outside this range is rejected.

Table 11 shows the results of the Kaiser–Meyer–Olkin (KMO) measure of sampling adequacy and Bartlett’s test of sphericity. More importantly, we consider the KMO value, as seen in Table 11. The accepted minimum KMO range of values is 0.5 minimum, which is fairly accepted, and 0.9 maximum, which is a perfect KMO value. Here, we have a KMO value of 0.859, which shows that our data are highly acceptable.

**Table 11:** The Kaiser–Meyer–Olkin (KMO) and Bartlett’s test results.

KMO or Bartlett’s Test		
**Kaiser–Meyer–Olkin measure of sampling adequacy**	0.859	
**Bartlett’s test of sphericity**	Approx. Chi-Square	7616.448
	df	276
	Sig.	>0.0001

The degree of freedom (df) shows a value of 276, which depicts a high chance of rejecting the null hypothesis (Ho). The level of significance (sig.) is less than 0.0001 which relates to a higher chance of rejecting Ho. The communalities table in Table 12 explains the level of acceptance across all variables, where the acceptable standard value of extraction is 0.3 and above. Thus, Table 12 clearly shows that all variables are acceptable for this research.

**Table 12:** Communalities table.

Communalities		
	Initial	Extraction
**SM use**	1.000	0.522
**Info use**	1.000	0.651
**IT SM use**	1.000	0.621
**SM as evolving tool**	1.000	0.666
**SM use for LG ePart**	1.000	0.548
**Govt. trust**	1.000	0.458
**Inc eGov dev**	1.000	0.642
**Bad attitudes of leaders**	1.000	0.525
**SM penetration rate**	1.000	0.568
**SM types**	1.000	0.550
**Prefer Twitter**	1.000	0.648
**Prefer Facebook**	1.000	0.417
**Prefer Instagram**	1.000	0.589
**Prefer Telegram**	1.000	0.653
**Prefer WhatsApp**	1.000	0.601
**Another effective SM**	1.000	0.687
**Twitter use by LG**	1.000	0.553
**Facebook use by LG**	1.000	0.457
**Instagram use by LG**	1.000	0.645
**Telegram use by LG**	1.000	0.601
**WhatsApp use by LG**	1.000	0.532
**Use of another SM by LG**	1.000	0.669
**SM inc e-participation**	1.000	0.490
**More effort made by LG**	1.000	0.623
Extraction method: Principal Component Analysis		

**Fig 3:**
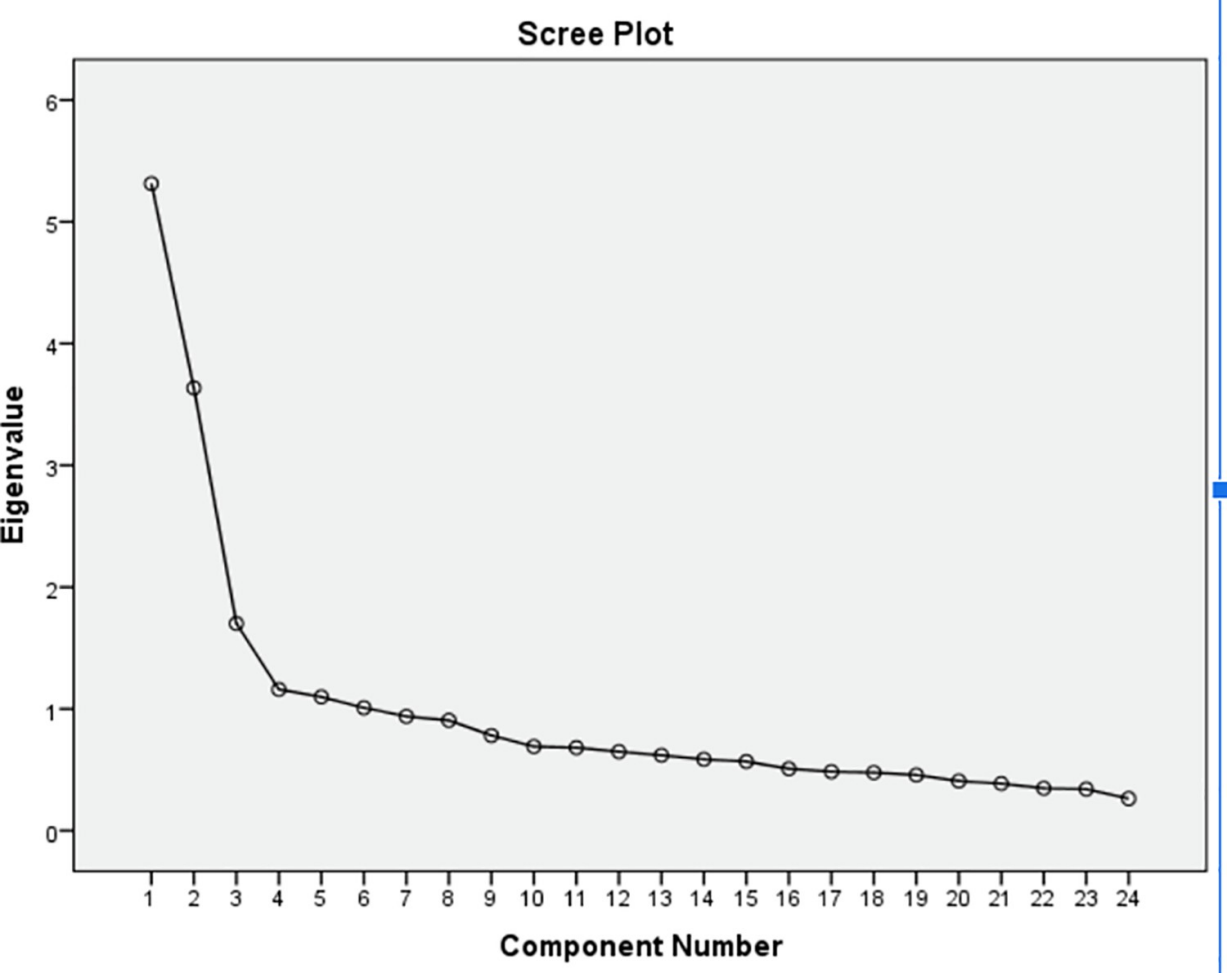
Scree plot.

Furthermore, we use the scree plot in Fig 3 to observe the Total Variance Explained in Table 13.

**Table 13:** Total variance explained for variables.

Total Variance Explained									
Component	Initial Eigenvalues			Extraction Sums of Squared Loadings			Rotation Sums of Squared Loadings		
	Total	% of Variance	Cumulative %	Total	% of Variance	Cumulative %	Total	% of Variance	Cumulative %
1	5.313	22.138	22.138	5.313	22.138	22.138	3.523	14.677	14.677
2	3.635	15.146	37.284	3.635	15.146	37.284	3.140	13.084	27.762
3	1.701	7.088	44.372	1.701	7.088	44.372	2.488	10.365	38.127
4	1.159	4.831	49.204	1.159	4.831	49.204	1.984	8.267	46.394
5	1.098	4.575	53.778	1.098	4.575	53.778	1.485	6.188	52.582
6	1.009	4.205	57.983	1.009	4.205	57.983	1.296	5.401	57.983
7	0.939	3.912	61.895						
8	0.906	3.775	65.670						
9	0.781	3.254	68.923						
10	0.691	2.878	71.802						
11	0.681	2.838	74.639						
12	0.648	2.702	77.341						
13	0.618	2.577	79.918						
14	0.586	2.440	82.358						
15	0.567	2.364	84.722						
16	0.508	2.115	86.837						
17	0.484	2.016	88.853						
18	0.476	1.985	90.839						
19	0.456	1.901	92.740						
20	0.407	1.696	94.436						
21	0.386	1.608	96.044						
22	0.347	1.446	97.490						
23	0.340	1.417	98.907						
24	0.262	1.093	100.000						

The eigenvalue represents the total variance that can be attributed to a variable. It is also known as the characteristic value. The eigenvalue is set to 1. Thus, only variables above an eigenvalue of 1 on the scree plot will be considered in the Total Variance table. Only six components cross the value of 1 on the scree plot. Therefore, the cumulative frequency of the six components in Table 14 is 57.983 percent, which is well above the minimum accepted value of 50 percent.

**Table 14:** Rotated component matrix for factors loaded together.

Rotated Component Matrix						
	Component					
	1	2	3	4	5	6
**SM use**	−0.393	−0.049	−0.204	**0.545**	−0.032	0.159
**Info use**	0.061	0.129	0.107	0.106	−0.026	0.779
**IT SM use**	**0.771**	−0.084	−0.032	0.062	0.083	0.082
**SM as evolving tool**	**0.788**	−0.168	0.092	−0.027	0.090	−0.014
**SM use for LG ePart**	**0.684**	0.025	0.107	0.023	0.242	−0.095
**Govt. trust**	0.293	0.342	0.097	−0.027	0.481	−0.119
**Inc eGov dev**	0.039	0.176	0.185	−0.035	**0.735**	0.184
**Bad attitudes of leaders**	0.205	−0.180	−0.079	0.065	**0.663**	−0.021
**SM penetration rate**	0.003	0.255	0.256	−0.153	0.351	**0.539**
**SM types**	**0.619**	0.003	0.186	−0.328	0.154	0.033
**Prefer Twitter**	0.205	**0.739**	−0.174	−0.157	0.040	0.050
**Prefer Facebook**	0.081	0.254	**0.575**	−0.110	0.054	−0.022
**Prefer Instagram**	−0.106	**0.660**	0.375	0.037	−0.011	−0.016
**Prefer Telegram**	−0.097	**0.649**	0.460	−0.032	0.037	−0.094
**Prefer WhatsApp**	0.319	0.051	**0.704**	−0.005	0.006	0.019
**Another effective SM**	−0.005	−0.040	−0.071	**0.822**	−0.016	−0.062
**Twitter use by LG**	0.102	**0.653**	0.038	0.025	−0.016	0.339
**Facebook use by LG**	0.071	0.182	**0.575**	−0.113	0.146	0.235
**Instagram use by LG**	−0.184	**0.671**	0.342	−0.016	0.043	0.204
**Telegram use by LG**	−0.136	**0.650**	0.358	−0.022	0.141	0.111
**WhatsApp use by LG**	0.211	0.143	**0.648**	−0.142	0.010	0.162
**Use of another SM by LG**	−0.150	−0.020	−0.064	**0.800**	0.044	0.021
**SM inc e-participation**	**0.636**	0.106	0.112	−0.150	−0.016	0.197
**More effort made by LG**	**0.676**	0.066	0.191	−0.349	−0.017	−0.055
Extraction method: Principal Component Analysis. Rotation method: Varimax with Kaiser normalization. a. Rotation converged in 8 iterations.						

Note: acceptable values of >0.5 are written in bold.

Finally, in Table 14, the Rotated Component Matrix shows how the components of variables are loaded together; this means the two or more variables that are loaded together can be categorized on the same construct.

Varimax orthogonal rotation was used in this factor analysis, which converged in eight iterations as seen in Table 14 Ideally, values that are closer to each other will load together for convergence validity, but too-low values like those below 0.5 are normally not considered. As we see in Table 6, the variables with social media (SM) use load together strongly (0.771, 0.788, and 0.684). Most usage of Twitter, Instagram, and Telegram also loads together, as does preference for SM channels (between 0.739 and 0.649). Another good loading is the increase in e-governance development and bad attitudes of leaders (0.735 and 0.663), as well as the loading together of “social media, increased participation” and “more effort in my local government” (0.636 and 0.676).

The Paired Sample Statistics for both e-participation and most Twitter users are shown in Table 15 above showing a standard deviation of 0.61261 and 1.317 respectively.

**Table 15:** Paired samples statistics of e-participation and most Twitter users.

Paired Samples Statistics							
	Mean	N		Std. Deviation	Std. Error Mean
Pair 1	E-participation on SM	3.3986	1000	0.61261	0.01937
	Twitter use by LG	3.36	1000	1.317	0.042

## Discussion

This study as one of its kind dives into the major issue of preferential participation through social media and the effect it has on governance. Some related studies whose focus was on examining social media governance over the years by different authors [11,13,53] have not pointed out the area of valuing preferences which are relatively important in encouraging participation and possibly also examining the factors influencing e-participatory governance, especially in the social media age where interaction and reactions are valued. This study highlights individuals’ preferences in social media e-participatory government adoption and implementation. In response to the first research question (RQ1), It was shown that the majority of Nigerian citizens prefer the use of social media for e-participatory governance, which will encourage more willingness to participate. It was also discovered that there is very low awareness and adoption of any form of e-government platform that encourages e-participation at the local government level of Nigeria [22]. Over time, e-participatory governance has been limited through various available platforms adopted without maximizing the ease social media might bring to e-participation and e-governance at large [45,54]. This shows the limitation in e-government development from the local government level since research has proven that citizens will love to participate in e-government initiatives provided the preference is put into consideration [34,45]. These findings highlight the degree to which social media and politics are intertwined globally. This study brought light to the fact that young people control and own a substantial portion of the social media examined which validates the veracity of their political interest. As far as interested youth are concerned, this is a key factor to consider in the process of elections and other political involvement.

According to the distributed questionnaires and statistical analysis, the findings revealed an association between the use of social media and the rate of e-participation. It was observed that most of the citizens prefer to participate using a range of social media, with WhatsApp having the highest preference, followed by Facebook, and then, Twitter [55]. This provides a reasonable argument for RQ2. Approximately 47% of social media users preferred WhatsApp and Twitter, but 79% of all users were known to use Facebook (for reaching large audiences), which has the most user involvement in e-participation and governance [36,56,57]. Based on our hypothesis H2, As far as social media e-participatory governance is concerned, some of the available social media platforms do not support real-time interactions which are needed for proper communication. This study shows that many prefer WhatsApp for interaction but we cannot underestimate the limitations of WhatsApp as a social media interaction tool. Facebook and Twitter(X) on the other hand have a more advanced way of interaction with their ability to retain Information and it’s open for registered users to comment, share, and react. Twitter(X) is the most popular microblogging site which has engagement measures proven to have a drastic effect on governance [34,58]. The highly rated text-based postings the rate of speed at which information is conveyed, and the accommodation of a large volume of registered users, make it difficult for Twitter studies to be undermined.

The study also revealed an association between e-participation and government trust, to such an extent that the level of trust earned by the government and its representatives determines the level to which citizens develop an interest in participating and their levels of interaction with and involvement in governance, knowing that their contributions will be valued and implemented.

However, both associations (between social media use and e-participation, and between government trust and e-participation) were shown in Table 16 to have a statistically weak positive correlation of 0.066 and 0.231, respectively. The weakness in the positive correlation means the relationship between both variables is not yet very strong because the values are not close to 1.0. This is a bit of contradiction to a study by Alarabiat et al. [45], which revealed a high level of e-participation of male and female participants in an online and offline e-participation study. This contradiction could be a result of a high level of collaboration and involvement of females in offline social groups of governmental and non-governmental organizations. However, the weak positive correlation in this study indicates the inefficiency of strong technological prowess and the functioning database of media users in Nigeria. However, this low correlation between trust in government and e-participation could be simplified as a non-necessary determinant for citizens’ participation. It could be observed that with the low correlation between the two variables, i.e. trust in government and e-participation, Nigerian citizens will still want to participate if the preferred medium of e-participation is made available. Therefore, we can deduce that trust is not a determinant factor for e-participation.

**Table 16:** Correlation between e-participation and most Twitter use.

Paired Samples Correlations				
	N		Correlation	Sig.
Pair 1	E-participation via SM and Twitter use by LG	1000	0.066	0.037

According to Vicente et al. [54], the subsidization and availability of the Internet for social media access have contributed to huge e-participation in governance in some countries, such as Germany, France, the United States of America, and the United Kingdom. Conversely, in Nigeria, the unavailability of adequate tax fares for internet subscribers and the violation of due process have hampered the effectiveness of e-participation in governance [33].

Okundaye et al. [32] also confirmed the link between social media and e-participation, but in reality, the benefits of e-participation were not accrued. The authors further explained that due to challenges from stakeholders, the context of e-participation, the research methods, and the theoretical background, the results of e-participation were not discovered. This is almost in line with the observations in this study showing a high response from the majority of participants, but a weak positive correlation and a few impacts on e-participation.

In a study by Hofmann et al. [59], numerous analyses were performed on the issue of e-participation, but several of the findings did not reach a decision-making stage. However, their findings were based on a Filipino Facebook algorithm check using a lexicon-based model and unsupervised machine learning to classify the emotions of Filipinos on the proposed bill of the minimum age of criminal liability. Their study results showed that 42% of the emotions were negative, while 54% were positive, and the remaining 4% were neutral. Thus, they concluded that user engagement on social media comes with varied emotions, but the online presence of government stakeholders can promote positive emotions and e-participation from users. This study, however, recorded a weak positive correlation between e-participation and social media which practically resolved the pondering RQ4.

The provision of adequate responses to the research questions, which can be seen as a road map for efficient electronic participation on social media in managing our country’s political arena and governing structures, has been effective in meeting all of the study’s objectives. Based on interactions discovered through examining social media engagement tools and other e-government platforms, the study determined the most efficient tools for communication and online participation. The social media platform Twitter, with its characteristic wide user base, tweet volume, and high number of followers made up of young people, has demonstrated competitive strength and has been determined the most effective social media platform as a remedy for a hampered political, electoral, and administrative government process [58].

A high turnover rate from technology-based companies will be achieved if collaborative efforts from government stakeholders are approved via due process. Accessibility and mobile data subscriptions, if enhanced and subsidized, respectively, will enhance trust and increase e-participation both within the nation and in the diaspora. The association between social media and e-participation is still weak due to the inefficient strong technological prowess and functioning database of media users, especially at the grassroots level. Other factors of this weakness may include past political chaos, economic instability, improper collaborative efforts, and government injustice against citizens.

## Implication

This study provides relevant information in the governing sector in a way to subdue the limitations of communication, participation, and interaction between the government, stakeholders, and citizens of Nigeria and the world at Large. It provides information about the level of awareness and adoption of e-government initiative platforms at the local government levels of Nigeria. It also identifies key factors that influence the adoption and successful implementation of e-government at the local level in Nigeria, such as technological infrastructure, public trust, and governmental support [60,61]. These insights can help in developing more comprehensive models of e-government adoption. Theoretically, it advances e-government theory by providing insights specific to Nigeria, refining adoption models, and contributing to governance theories on transparency and citizen engagement [62,63]. Practically, the study informs policymakers with recommendations to improve ICT infrastructure, staff training, and digital literacy. It offers guidelines for local governments to enhance service delivery, efficiency, and reduce corruption, leading to greater public satisfaction and trust. Additionally, the research emphasizes the need for capacity building among government employees and strategies for increasing citizen engagement through e-government platforms and social media. It also provides a benchmark for other local governments, presenting best practices adaptable to similar contexts.

## Conclusions

The study applies a quantitative research methodology, collecting and analyzing data from 1000 samples to examine the most preferred applications or platforms for communication and interaction through e-participation among citizens, stakeholders, and local government entities. The findings reveal a relatively low level of awareness, and adoption of e-government initiatives which could enable e-participation by citizens. A high level of interest in e-participation through various preferred social media platforms was discovered, also the correlation between government trust and e-participation using these platforms is minimal. The study aims to improve and recommend preferred e-participation tools for citizens and government parastatals, as well as identify factors driving e-participation in the Nigerian local government context. With the geometric rate at which social media is penetrating the nukes and crannies of the rural areas, especially in Nigeria, the government could utilize social media more effectively in reaching out to its citizens by encouraging social media e-participation with a consciousness of prioritizing citizens’ preference in participation. Twitter(X) as the most popular microblogging site has also provided a more sophisticated means of communication with reactions that can be analyzed to understand the citizen’s participation and interest in government activities and initiatives.

### Limitations and recommendations

The extent to which the chosen samples were covered is a significant study constraint. Firstly, the study could not fully involve some geopolitical zones because of the prevailing insecurities and agitations. Additionally, the perceived bias and socio-cultural orientation of respondents in these regions could result in improper statistics and misinformation in the research protocols.

Secondly, due to the inaccessibility of internet connection in many parts of the north-west region of the country, the researchers could not generate information from respondents in those regions electronically. Hence, to address this challenge, the researchers employed the method of offline paper-based questionnaires acquired by printing out the same questionnaires administered using the online Google form. This method helped the researchers to achieve the objective of the study.

Thirdly, conventional literacy disparity affected respondent–research interactions. In most rural areas of all the geopolitical zones of the country, the researchers encountered communication hitches and had to employ the services of an interpreter in many parts of the country. These interpreters translated from English to languages such as Hausa, Yoruba, Ibibio, Fulfulde, Kanuri, Ebira, and Vernacular. Hence, due to the literacy level in these areas, this study observes almost zero percent e-participation in governance.

As social media, like Twitter, expands with general technology usage, which, in turn, has been accompanied by online government advocacy or electronic governance in recent years, it is impossible to overstate the great eclipses of contemporary technology. Increasing citizen understanding of how internet services provided by the government operate is essential, among other ideas. Finding out how effective e-governance occurs through social media is one of the main objectives. Proper public management relies on partnerships between the public and commercial sectors. Web navigation abilities and exposure to e-governance both improve as the use of the Internet for everyday communication increases.

It is worth highlighting the impact of government stakeholders on the success and progress of e-participation in e-governance. A lack of government support could result in the stagnation of political resurgence and a lack of personal interest from citizens [35,37,54]. Therefore, the gap between the leaders and the leaders should be closed or reduced so that the level of trust, which was discovered to be associated with e-participation in this study, will increase exponentially.

The evidence of the interaction between citizens and the polity cannot be over-emphasized. The goodwill of citizens is integral to their participation in governance and maintaining a consistent rapport with the government. This intention will produce a positive result for e-participation if relevant stakeholders maximize the huge potential of young people’s interest in governance with guided policies, terms, and conditions to assist the departments, ministries, and parastatals with digital communications, and thus, effectively manage the decision-making processes that will unite the nation and move it forward.
